# Common variable immunodeficiency associated with microdeletion of chromosome 1q42.1-q42.3 and inositol 1,4,5-trisphosphate kinase B (ITPKB) deficiency

**DOI:** 10.1038/cti.2015.41

**Published:** 2016-01-22

**Authors:** Ankmalika G Louis, Leman Yel, Jia N Cao, Sudhanshu Agrawal, Sudhir Gupta

**Affiliations:** 1 Programs in Primary Immunodeficiency and Human Aging, Division of Basic and Clinical Immunology, Department of Medicine, University of California, Irvine, CA, USA

## Abstract

Common variable immunodeficiency (CVID) is a heterogenous disorder characterized by hypogammaglobulinemia and impaired specific antibody response and increased susceptibility to infections, autoimmunity and malignancies. A number of gene mutations, including *ICOS*, *TACI* and *BAFF-R*, and *CD19*, *CD20*, *CD21*, *CD81*, *MSH5* and *LRBA* have been described; however, they account for approximately 20–25% of total cases of CVID. In this study, we report a patient with CVID with an intrinsic microdeletion of chromosome 1q42.1-42.3, where gene for inositol 1,3,4, trisphosphate kinase β (*ITPKB*) is localized. ITPKB has an important role in the development, survival and function of B cells. In this subject, the expression of *ITPKB* mRNA as well as ITKPB protein was significantly reduced. The sequencing of *ITPKB* gene revealed three variants, two of them were missense variants and third was a synonymous variant; the significance of each of them in relation to CVID is discussed. This case suggests that a deficiency of *ITPKB* may have a role in CVID.

Common variable immunodeficiency (CVID) is a heterogeneous disorder of unknown etiology characterized by decreased immunoglobulin G (IgG) and additional Ig isotype(s) and impaired specific antibody responses; they often present with increased susceptibility to recurrent infections, autoimmune diseases and malignant disorders.^1, 2, 3, 4, 5, 6^ In contrast to monogenetic antibody deficiency disorder such as x-linked agammaglobulinemia, a single common gene defect has not been identified for CVID. Mutations in a number of genes associated with B-cell development and functions, that is, *ICOS*, *TACI* and *BAFF-R*, and *CD19*, *CD20*, *CD21*, *CD81, MSH5* and *LRBA* have been observed in patients with CVID.^7, 8, 9, 10, 11, 12, 13, 14, 15, 16, 17^ However, they contribute to approximately 20% of all cases of CVID. Therefore, there is a need to identify additional gene defects associated with CVID.

To identify a potential candidate gene, we investigated a patient with CVID who had a microdeletion of chromosome 1q42.1-q42.3. In this subregion, genes were reviewed and the gene for inositol 1,4,5-trisphosphate 3 kinase β (ITPKB) was identified as a potential responsible gene for the patient's immunological defects. ITPKB is one of three inositol 1,4,5-trisphosphate (IP3) kinase isoforms that is ubiquitously expressed and has an important role in the development, survival and functions of various hematopoietic cells.^18, 19, 20, 21, 22, 23, 24, 25, 26, 27^ ITP3KB phosphorylates IP3 to inositol 1,3,4,5-tetrakisphosphate (IP4) and control signal transduction in hematopoietic cells.^23, 25, 27^ *ITP3KB* gene has been implicated as a candidate gene in a number of human disorders, including Alzheimer's disease, multiple sclerosis and malignant melanoma.^28, 29, 30^ An association of ITPKB deficiency in CVID has not been previously described.

Herein we report a 26-year-old male patient with CVID and a constellation of findings, that is, schizophrenia, developmental delay, seizures, dystonia and hyperkeratotic eczematous dermatitis, who has an interstitial deletion of chromosome 1q42.1-42.3. This subregion carries the responsible genes for schizophrenia, seizure disorder, ectodermal dysplasia and dystonia as well as the gene for ITPKB. We believe that microdeletion of 1q42.1-42.3 resulting in haploinsufficiency of this subregion at least contributes to, if not causing, this patient's clinical findings.

## Results

### Case report

A 26-year-old Caucasian male patient was evaluated for recurrent skin infections due to *Staphylococcus aureus*. The papulo-pustular skin lesions started on his face, trunk and extremities in his early twenties. Multiple cultures were remarkable for staphylococcus, including methicillin-resistant *S. aureus*. He required multiple courses of antibiotics along with incision and drainage for several deep skin abscesses. The patient experienced recurrent ear infections as a child and was diagnosed as having allergic rhinitis, eczema, schizophrenia, dystonia, seizure disorder and developmental delay. Physical examination revealed a diffuse erythematous rash and multiple small papulo-pustular lesions on the face, trunk and extremities and hyperkaratotic eczematous dermatitis.

### Chromosome analysis

**Figure 1:**
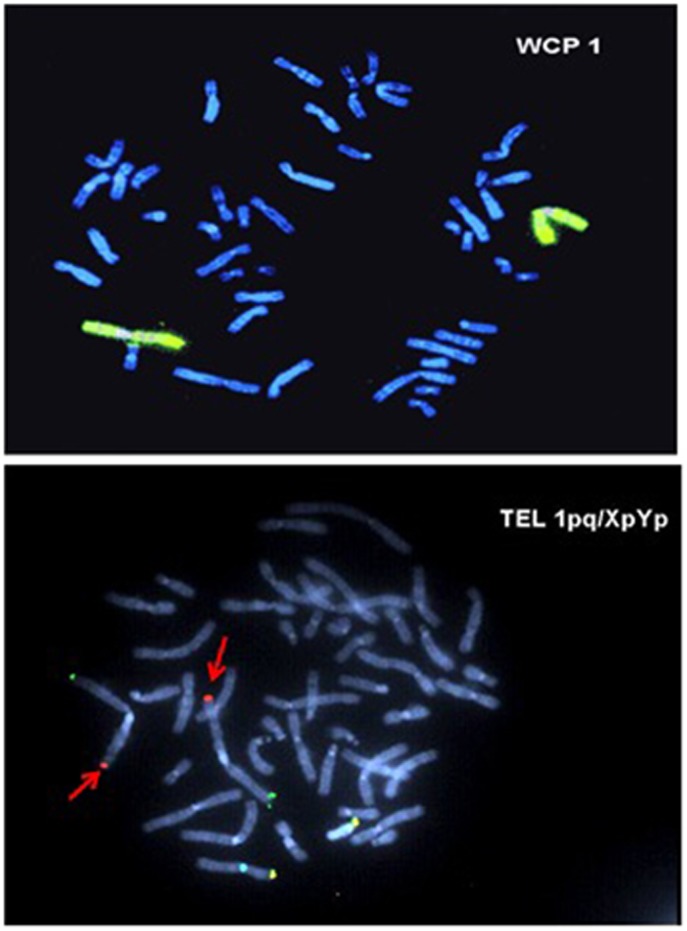
Fluorescent *in situ* hybridization analysis showing heterozygotic microdeletion of chromosome 1q42.1-42.3 (arrows). The top panel shows the wcp1 staining, and the bottom panel shows the presence of subtelomere region.

Cytogenetic and fluorescent *in situ* hybridization analysis were performed because patient has microcephaly and developmental delay. Cytogenetic analysis shows additional material attached to the long arm of chromosome 1 (G banding): 46,XY,add (1)(q42.1). This may represent a deletion or an interchromosomal rearrangement. Fluorescent *in situ* hybridization analysis using whole chromosome paint 1 (wcp1) probe and subtelomere probe specific for chromosome 1q (D1S3738) revealed the presence of hybridization signal over the entire abnormal chromosome 1 (Figure 1, top panel) and the presence of subtelomere region (Figure 1, bottom panel). The abnormality is therefore interpreted to represent an interstitial deletion of the long arm of chromosome 1 (ish del (1)(wcp1+, D1S3738+).

### Immunological studies

Immunological features of the patient are shown in Table 1. Serum levels of IgG and IgA were decreased and IgM levels were normal. The patient did not respond to any of the 14 serotypes of *Streptococcus pneumoniae* following Pneumovax-23 vaccination. Lymphocyte subsets revealed decreased number of CD3+ T cells, decreased numbers of NK cells and normal numbers of B cells. Density of T-cell receptor on lymphocytes and the expression of HLA-DR were comparable to those of the control. Lymphocyte proliferation in responses to antigens, including mumps, tetanus, purified protein derivative and *Candida albicans*, were almost absent; however, response to mitogens phytohemagglutinin, concanavalin A and pokeweed mitogen were moderately reduced.

**Figure 2:**
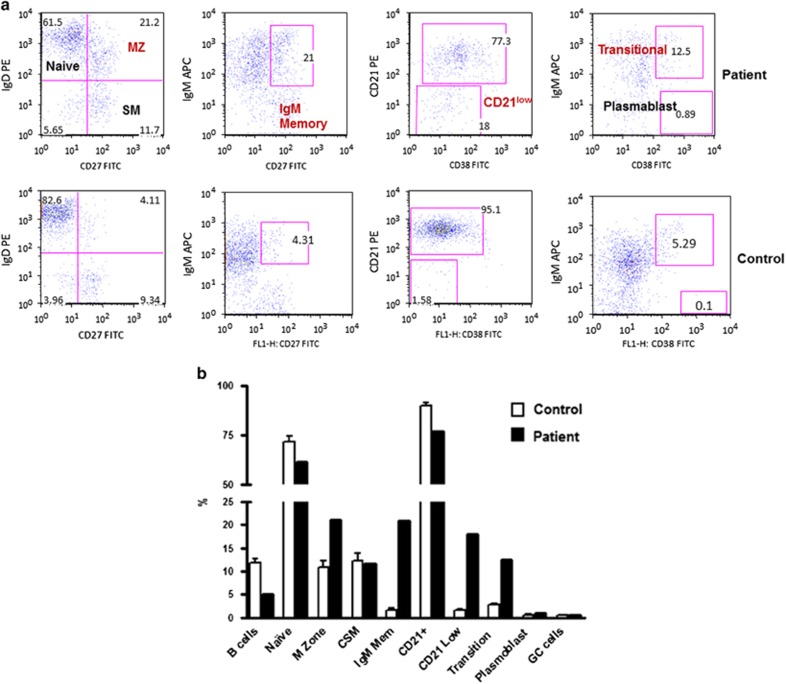
A flow cyometric graph of B-cell subsets in a patient and a healthy control (**a**). (**b**) B-cell subsets in the patient as compared with 20 healthy controls. Marginal zone (MZ), transitional B cells, IgM memory B cells and CD21^lo^ B cells were increased.

Further analysis of B-cell subsets revealed increased proportions of marginal zone B cells, transitional B cells, IgM memory B cells and CD21^low^ B cells (Figure 2).

**Figure 3:**
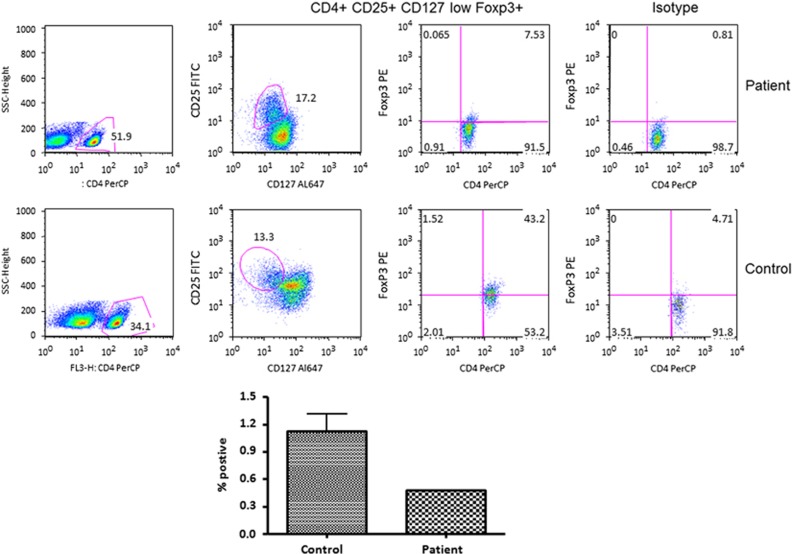
A flow cytometric graph of Treg cells (top panel). Cells were gated on CD4+ T cells and then examined for the expression of CD25, CD127 and FoxP3. Bottom panel shows the patient's Treg as compared with 20 healthy controls. Treg were calculated as percent positive of total foxp3 cells in CD4 population=(CD4+) × (CD25 high CD127 low) × (foxp3+ve)/(10 000). In the patient, CD4+CD25+CD127^lo^FoxP3+ Treg cells were markedly reduced as compared with control.

FoxP3+CD25+CD127^lo^CD4+ Treg (regulatory T) cells were markedly decreased as compared with control. Patient has 0.6% and control has 1.9%. Bar graph is the value of patient and 20 healthy control subjects (Figure 3).

#### Respiratory burst

Nitroblue tetrazolium test was normal. In the patient, oxidative burst using dihydrorhodamine 123 (DHR123) by flow cytometry was impaired compared with control (Table 1).

#### ITPKB gene expression

**Figure 4:**
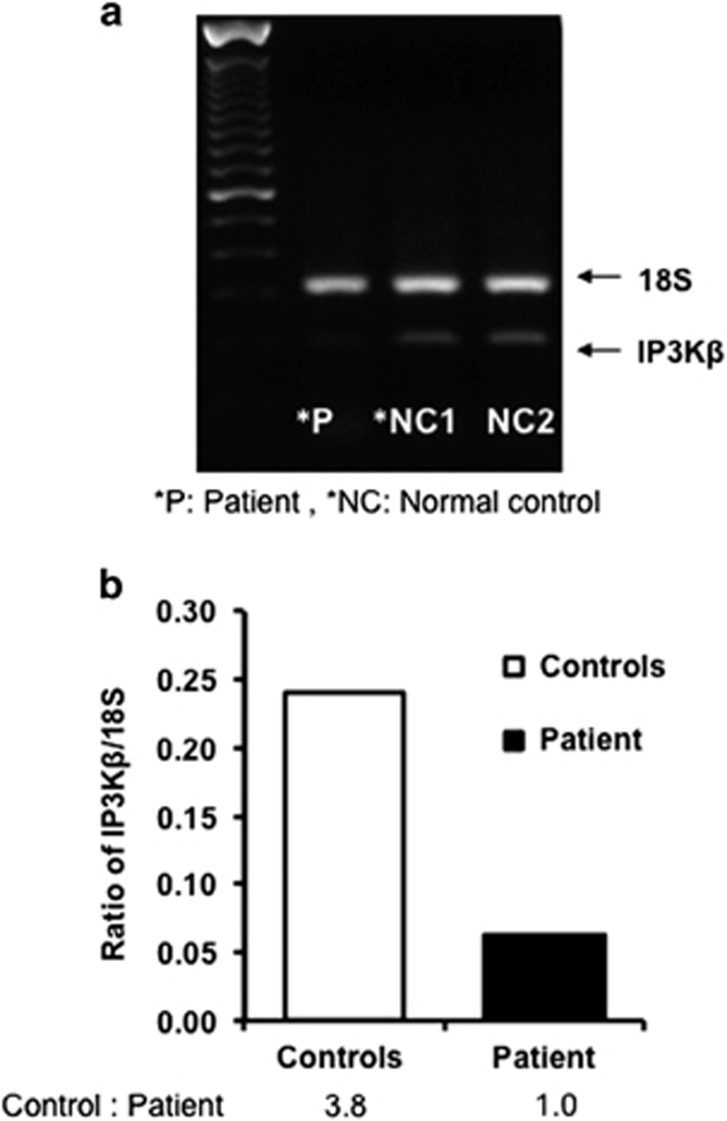
RT-PCR for *ITPKB* mRNA. RT-PCR was performed on activated PBMCs (anti-CD3 for 6 h) from the patient and two healthy controls using specific primers and *ACTB* as a housekeeping gene. Top panel (**a**) shows a gel and the bottom panel (**b**) show quantitation of gels by densitometer. A significant decreased in *ITPKB* mRNA was observed.

**Figure 5:**
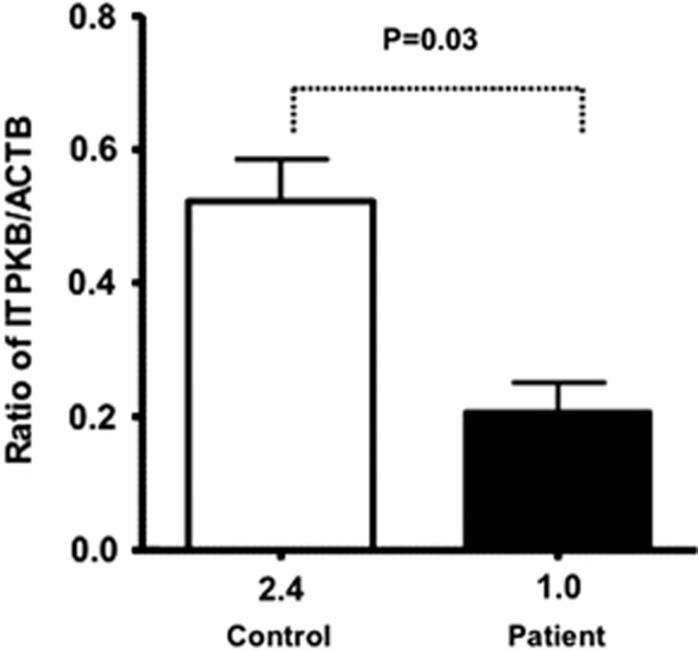
qRT-PCR for *ITPKB* mRNA. Quantitative RT-PCR was performed on freshly isolated PBMCs using specific primers. Three different samples from the patient were obtained at different time intervals. A significantly reduced *ITPKB* mRNA was observed.

Both reverse transcriptase PCR (RT-PCR) from peripheral blood mononuclear cells (PBMCs; Figure 4) and quantitative RT-PCR (qRT-PCR) from PBMCs (Figure 5) showed that gene expression of *ITPKB* was significantly decreased (3.8-fold for RT-PCR and 2.4-fold for qRT-PCR) in the patient as compared with control.

#### ITPKB protein expression

**Figure 6:**
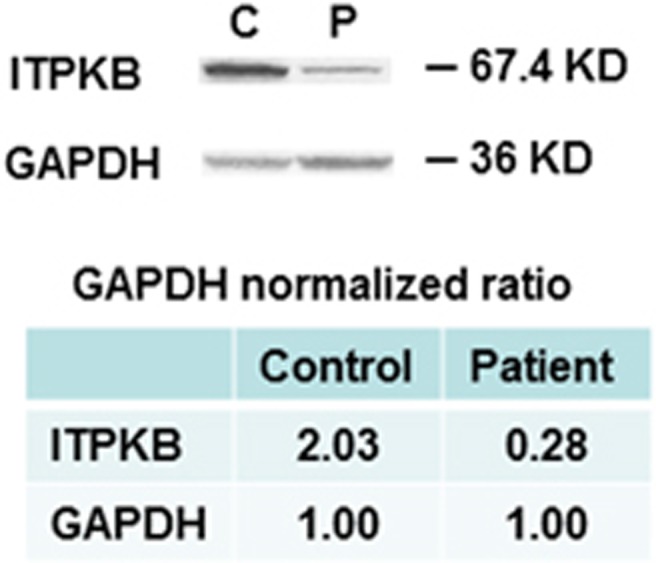
ITPKB protein expression. Western blotting was performed on protein extracted from mononuclear cells using specific anti-ITPKB antibody and GAPDH as internal loading control. Top panel shows gel and bottom panel shows quantitation of gels measured by densitometry and expressed as the ratio of ITPKB/GAPDH, which show markedly deceased expression of ITPKB in the patient.

ITPKB protein expression was measured by western blotting using rabbit anti-ITPKB-specific antibody and GAPDH (glyceraldehyde 3-phosphate dehydrogenase) as an internal loading control. Each band volume was measured by densitometry and the ratio of ITPKB/GAPDH is presented. Figure 6 shows that the ITPKB expression was markedly decreased in the patient (P) as compared with healthy control (C).

#### ITPKB gene sequence

**Figure 7:**
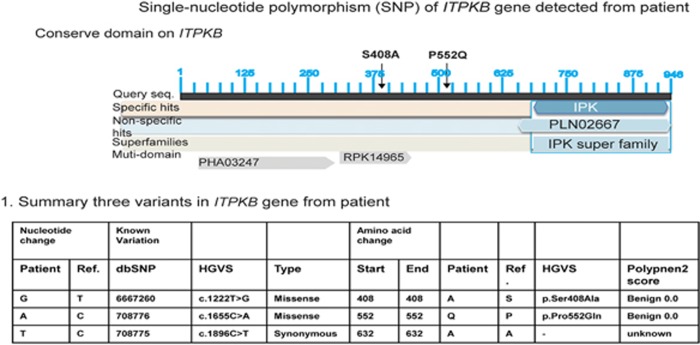
Three variants of *ITPKB* gene and their locations.

We sequenced the DNA of eight exons and their adjacent introns of the *ITPKB* gene from patient's genomic DNA. Three variances in this gene were identified; two missense variants: (1) S (TCG)→A (GCC), (2) P (CCG)→Q (CAG), and a third synonymous variant A (GC**C**)→A (GC**T**). The graphic conserved domain on *ITPKB* gene and the location of two missense variants are shown in Figure 7.

Their allelic frequency in general population is shown in Table 2. Two missense variants are common in general population and are considered benign. Third variant is synonymous.

## Discussion

CVID, excluding selective IgA deficiency, is the most common primary immunodeficiency in adults, with a prevalence of approximately 1 in 10 000–50 000.^1^ It is a heterogenous disorder characterized by recurrent infections and an increased risk for autoimmune and malignant disorders.^3, 4, 5^ Most cases are sporadic in origin but autosomal-dominant and autosomal-recessive inheritance patterns have been recognized in some families.^31, 32^ Several family studies have also identified susceptibility loci in the major histocompatability complex region.^33, 34, 35^ The immune system defect is characterized by hypogammaglobulinemia, impaired specific antibody response and impaired cell-mediated responses in up to 50% of patients. T-cell abnormalities may include CD4+ lymphopenia, diminished lymphoproliferative responses to antigens and mitogens and impaired cytokine production.^1, 2, 3, 4, 5, 6, 32, 36^ Our patient also displayed low CD3+ T cells and markedly impaired response T-cell responses to recall antigens, suggesting a combined T- and B-cell defects. Furthermore, our patient has severe deficiency of Treg cells. Similar deficiency of Treg has been reported in patients with CVID.^37, 38, 39, 40^ Although some of the molecular defects in CVID have been identified, including mutations of *ICOS*, *TACI* and *BAFF-R*, and *CD19*, *CD20*, *CD21*, *CD81*, *MSH4* and *LRBA* genes,^7, 8, 9, 10, 11, 12, 13, 14, 15, 16, 17^ the genetic basis for this disorder still remains to be elucidated. In view of the heterogenous clinical features and absence of a single gene defect, CVID appears to represent different genetic disorders that have yet to be characterized.

The present case provides an opportunity to assess the genotype–phenotype relationship in a patient with haploinsufficiency of chromosome 1q42.1-42.3 and CVID. Chromosome 1 is the largest human chromosome. Chromosome 1 spans about 249 million nucleotide base pairs and represents about 8% of the total DNA in human cells. Chromosome 1 is currently thought to have 4316 genes. Using the Online Mendelian Inheritance in Man (OMIM) database, the individual genes located on chromosome 1q42.1-42.3 were reviewed, and *ITPKB* was identified as a potential responsible gene for the immunological defect in CVID.

The mammalian IP3 kinases consist of a family of three isoforms, A, B and C.^18, 19^ These three isoforms share a relatively well-conserved catalytic domain and specifically convert IP3 to IP4. An isoform (gene localized on chromosome 15q14) is associated with cytoskeleton, where C isoform appears exclusively localized to cytoplasm. In contrast, B isoform (ITPKB) is associated with plasma membrane, cytoskeleton and found in the endoplasmic reticulum.^21^ More recently, Nalaskowski *et al.*^41^ have identified subnuclear localization of ITPKB. They showed that ITPKB is enriched at nuclear invagination. They also showed that ITPKB is involved in the regulation of both cytoplasmic and nuclear calcium signaling.

ITPKB is a ubiquitously expressed enzyme that has an important role in the development and functions of hematopoietic cells. In the T cells, ITPKB appears to have a role in thymic selection and T-cell calcium signaling.^20^ In the B cells, ITPKB regulates B-cell selection, tolerance, survival and negatively regulate B-cell signaling.^22, 23, 24, 25^ ITPKB also has a role in myelopoiesis and negatively regulates neutrophil signaling.^26, 27^

In mice, disruption of the *ITPKB* gene results in decreased IP4 and block in thymocyte-positive selection and to a severe T-cell deficiency. A mouse strain with nonsense mutation of *ITPKB* gene shows a complete block in positive selection of T lymphocytes at double-positive stage in the thymus and impaired T-cell receptor signaling.^23^ Our patient had only modest decrease in CD3+ T cells and in mitogen responses; however, his T-cell responses to antigens were severely compromised. This modest quantitative and functional T-cell deficiency in our patient may explain lack of opportunistic infection. Mice lacking *ITPKB* shows normal B-cell development in the bone marrow but low number of splenic B cells.^25^ *ITPKB−/−* B cells displayed impaired response to BCR signal, but normal response to CD40 cross-linking and LPS, therefore resembling tolerant B cells. Furthermore, these mice show impaired IgG3 response to T-independent antigens. In CVID, various alterations in B-cell differentiation and impaired antibody responses have been reported.^42, 43^ These include expansion of CD21^lo^ and deficiency of switched memory B cells. Our patient shows markedly impaired response to T-independent pneumococcal polysaccharide antigens and expansion of CD21^lo^ B cells; however, switched memory B cells were comparable to healthy controls.

In the patient, both RT-PCR and qRT-PCR showed significantly (*P*<0.03) reduced expression of *ITPKB* mRNA. Furthermore, ITPKB at the protein level was also markedly decreased.

As gene sequencing revealed three variants in *ITPKB* gene in our patient, do these gene variants have a role in immunological defects in CVID? As two missense variants are considered benign and are frequently observed in the general population, it is unlikely that they would be contributing to CVID. The third variant is a synonymous. A synonymous substitution, often called a silent substitution, is a substitution of one base for another in an exon of a gene coding for a protein, such that the produced amino-acid sequence is not modified. Synonymous substitutions affecting noncoding DNA are often considered silent; however, this is not always the case.^44, 45, 46, 47, 48^ Synonymous changes may not be neutral because certain codons are translated more efficiently than others. Synonymous mutations can affect transcription, splicing, mRNA transport and translation, any of which could render the synonymous mutation non-silent.^46^ Another reason why synonymous changes are not always neutral is the fact that exon sequences close to exon–intron borders function as RNA splicing signals. When the splicing signal is destroyed by a synonymous mutation, the exon does not appear in the final protein. This results in a truncated protein.^49, 50^ It is possible that the synonymous variant in our patient may be contributing to the decreased transcription of *ITPKB* gene, and decreased expression of ITPKB at the protein level, which would be mostly due to haploinsufficiency as a result of chromosomal deletion. As patient does not know whereabouts of his parents, we could not establish whether deletion was a *de novo* deletion.

In addition to T-cell development, the IP3–IP4 calcium pathway has been shown to be important for other immune functions, including proliferation, cytokine production, cytotoxicity and respiratory burst. The formation of oxygen-free radicals has been shown to be in part dependent on the onset and magnitude of the cytosolic calcium signal. The activation of neutrophil oxidase is dependent on free cytosolic calcium.

In this study, we have shown that microdeletion of chromosome 1q42.1-42.3 results in decreased expression of *ITPKB* gene. Given the importance of calcium signaling for generation of reactive oxygen species as well as the role of ITPKB in neutrophil functions and impaired DHR and impaired T-cell functions in our patient, we considered that defective calcium signaling in neutrophils and lymphocytes can result in abnormal respiratory burst and impaired humoral and cell-mediated responses. With the exception of CVID, the clinical and immunological findings for the present case were not consistent for any other well-defined disorders. We considered the possibility of Chediak–Higashi syndrome because the gene for this disorder, *Lyst*, resides on the 1q42.1-42.3 segment and because our patient presented with pyogenic infections and seizures, which are consistent features of this syndrome. However, other important diagnositic features, such as neutrophil giant granules and partial oculocutaneous albinism were not present. We also entertained the possibility of chronic granulomatous disease (CGD) as these patients present with recurrent infections and abnormal respiratory burst functions. However, patients with CGD have a much more profound defect in respiratory burst. Diagnostic criteria for CGD requires respiratory burst to be <5% of control, which was not the case of our patient. Furthermore, other consistent features of CGD, including granulomas and hepatosplenomegaly, were absent in our patient. It is unclear whether modest impairment of oxidative burst contributed to the susceptibility to staphylococcic skin infections observed in this instance.

In addition to CVID, this patient presented with a constellation of other clinical disorders, including, seizures, dystonia and schizophrenia; the susceptibility genes for each of these disorders have been identified and mapped to the q42.1-q42.3 subregion of chromosome 1 (OMIM). *DISC1* and *DISC2* genes for schizophrenia are localized at 1q42.1,^51^ and gene for myotonic dystrophy (*CDC42BPA*, *MRCA*, *PK-428*) localized to 1q42.11.^52, 53^ Although we did not investigate the expression of these genes, it is likely that haploinsufficiency of this subregion results in decreased expression of the responsible genes for those disorders.

We conclude that haploinsufficiency of chromosome 1q42.1-q42.3, resulting in ITPKB deficiency may be responsible for the impaired antibody and cell-mediated immune responses and also for the attenuated respiratory burst in neutrophils in our patient with CVID.

## Methods

### Subjects

PBMCs were isolated from the blood of patient and healthy subjects by Ficoll-hypaque density gradient (Mediatech, Inc., Manassas, VA, USA). Protocol was approved by the Human Subject Committee of the Institution Review Board (Human), University of California, Irvine, CA, USA. Informed consent was obtained from the patient.

For T-cell subsets, antibodies used were CD4 PerCP (peridinin chlorophyl protein) and CD8 PerCP, CD45RA APC (allophycocyanin), CCR7 FITC (fluorescein isothiocyanate), CD3 PerCP and CD278 (ICOS) PE (phycoerythrin). All antibodies were obtained from BD Pharmingen (San Jose, CA, USA).

### Chromosome analysis and fluorescent *in situ* hybridization

Cytogenetic analysis was performed using G banding (Genzyme Genetics, Pasadena, CA, USA). Fluorescent *in situ* hybridization was carried out by WCP1 probe and DIS3736 subtelomere probe specific for chromosome 1.

### B-cell subsets

B-cell subsets were analyzed on whole blood by incubating various combinations of monoclonal antibodies, including anti-human CD19 PerCP, anti-IgM APC, CD27 FITC, CD38 FITC, anti-IgD PE, CD21 PE, CD70 PE, CD27 APC, CD24 FITC, CD38 PE, CD183 PE (BD Pharmingen) and CD43 APC (Biolegend, San Diego, CA, USA) monoclonal antibodies, and corresponding isotypes. After staining, blood was lysed by 1 × lysing solution (BD Pharmingen) and washed with phosphate-buffered saline and analyzed. Flow cytometry was performed using FACSCalibur (Becton-Dickenson, San Jose, CA, USA) equipped with argon ion laser emitting at 488 nm (for FITC, PE and APC excitation). Forward and side scatters were used to gate and exclude cellular debris. Ten thousand cells were acquired and analyzed using the Flowjo software (Tree star Inc., Ashland, OR, USA).

### Detection of Treg cells

Cells were stained with CD4 PerCP, CD25 FITC and CD127 Alexa647 monoclonal antibodies and corresponding isotypes (BD Pharmingen), according to the manufacturer's protocol. Followed by Foxp3 intracellular staining with Foxp3 PE monoclonal antibody, isotype control (Mouse IgG1κ-PE) was used to evaluate nonspecific staining. Staining procedures was performed according to the manufacturer's recommendations. Treg cells were identified as CD4+CD25+CD127^Lo^Foxp3+ and analyzed with FACSCalibur (BD Biosciences, San Jose, CA, USA). Treg were calculated as percent positive of total foxp3 cells in CD4 population=(CD4+) × (CD25 high CD127 low) × (foxp3+ve)/(10 000).

### Respiratory oxidative burst in neutrophils

Oxidative burst was measured with DHR123 using flow cytometry. DHR123 is a nonfluorecent dye but it becomes highly fluorescent when oxidized by reactive oxygen species. Neutrophils were loaded with cell permeable DHR123 (Invitrogen, San Diego, CA, USA) at 37 °C for 15 min. The oxidative burst was induced by adding 100 ng of PMA (Sigma, St Louis, MO, USA) to DHR-loaded cells. After incubation at 37 °C for 15 min, red cells were lysed with BD FACS lysing solution, and increase in fluorescence associated with oxidation of DHR123 was analyzed on FACSCalibur using the cell quest software, and the mean fluorescence channel intensity (MFC number) were recorded.

#### Preparation of RNA and genomic DNA from the patient and controls

Total RNA was extracted from unactivated or activated PBMCs (CD3 1 μg ml^−1^ for 6 h) using a TRI Reagent Kit (Molecular Research Center Inc., Cincinnati, OH, USA), following the manufacturer's protocol. The integrity of intact total RNA was verified by measuring the ratio of rRNA OD (28S/18S) and the RNA integrity number with an Agilent 2100 Bioanalyzer (Agilent Technologies, Palo Alto, CA, USA). Genomic DNA was extracted from whole blood from the patient by using the PAXgene Blood DNA Kit (QIAGEN Inc., Valencia, CA, USA) following the manufacturer's protocol.

#### RT-PCR of ITPKB gene from activated PBMC RNA

RT-PCR was performed on RNA extracted from activated PBMCs from two healthy donors and the patient. One microgram of total RNA was reverse transcribed for patient and controls samples by using the SuperScript II reverse transcriptase from GIBCO BRL (Gathersburg, MD, USA) and a mix of oligo (dT) (100 ng per reaction) and random primers (100 ng per reaction) in a 20-μl total reaction volume at 45 °C for 50 min, according to the provided protocol. At the end of the RT reaction, the tubes were heated at 90 °C for 5 min to stop the reaction. A relative RT-PCR method using 18S as an internal standard (Ambion, Austin, TX, USA) was applied to investigate the expression of specific mRNAs for *ITPKB*. Primer sequences for *ITPKB* were (forward: 5′-AGCAGCCCTGGCCCTGTAAGATG-3′ reverse: 5′-ATGGGGAGAGGGGTGCAGAACAG-3′ GenBank accession no. NM_ 002221). These primers were purchased from Life Technology, GIBCO. In each PCR reaction, 18S ribosomal RNA was co-amplified with the target cDNA (mRNA) to serve as an internal standard and to allow correction for any differences in starting amounts of total RNA. For the 18S amplification, we used the Alternate 18S Internal Standards (Ambion), which yields a 324-bp product. The 18S primers were mixed with competimers at an optimized ratio 1:30, depending on the abundance of the target mRNA. Inclusion of 18S competimers was necessary to bring down the 18S signal, which allows its linear amplification to the same range as the co-amplified target mRNA (Relative RT-PCR Kit protocol, Ambion). The PCR reactions were carried out in the presence of 2 mm MgCl_2_ by using standard PCR buffer (GIBCO), 0.1 mM dNTP each, 1 μM specific primer set, 0.5 μM 18S primer–competimer mix and 0.75 unit of DNA *Taq* polymerase (GIBCO) in 25 μl total volume. A Perkin-Elmer DNA thermocycler (Norwalk, CT, USA) was used for PCR amplification, with 38 cycles of denaturation at 95 °C for 45 s, annealing at 55 °C for 45 s, extension at 70 °C for 45 s and extension for 10 min at 70 °C after last cycle. In all, 10 μl of PCR products were run on 2% agarose gels (Sigma) with TBE buffer, visualized with ethidium bromide and photographed. Signal quantification was conducted by laser-scanning densitometry. *ITPKB* mRNA signal was normalized to its corresponding 18S.

#### Real-time qRT-PCR of ITPKB gene from PBMC RNA

qRT-PCR was performed on RNA extracted from PBMCs from four healthy donors and from three separate samples donated by the patient on three different occasions. One microgram of total RNA was reverse transcribed to cDNA by using the High Capacity cDNA Reverse Transcription Kit (Life Technologies, Applied Biosystems, Grand Island, NY, USA) with random hexamers as primers following the manufacturer's instructions. The cDNA was amplified by PCR in a 20-μl reaction mixture containing 2 μl of 1 μM forward and reverse primers, 10 μl of SYBR Green 1 Master Mix (Roche Life Science, Indianapolis, IN, USA) and 4 μl of either cDNA diluted 10 × for *ITPKB*, cDNA diluted 300 × for *ACTB* or water as a negative control. Initial denaturation of DNA was carried out at 95 °C for 10 min; 40 amplification cycles were performed, each cycle consisting of denaturation (95 °C, 30 s) and annealing with extension (65 °C, 1 min). Each sample was amplified in triplicate, and results were normalized with *ACTB* gene expression as ‘housekeeper. A 4-log absolute standard curve dilution series was run using each primer pair at optimal concentration, and amplification efficiencies and slop were calculated. The fold changes of differential expression between healthy donors and the patient subject were calculated using the ratios of *ITPKB* to *ACTB*. Primers for *ITPKB* and *ACTB* were purchased from http://www.RealTimePrimers.com.

Statistical analysis was performed by Student's *t*-test.

#### Western blotting for ITPKB protein

PBMCs were lysed with lysis buffer (Cell Signaling, Danvers, MA, USA). Aliquots of cell lysates containing 50 μg of total protein were resolved by sodium dodecyl sulfate-polyacrylamide gel electrophoresis and transferred onto membranes (Millipore, Bedford, MA, USA) by electroblotting. The membranes were blocked for 1 h at room temperature in TBS-T buffer with 5% nonfat dried milk and incubated with 1μg ml^−1^ primary anti-ITPKB antibody (Sigma-Aldrich, St Louis, MO, USA) and anti-GAPDH antibody as loading control (abcam Cat. No: ab8245, Cambridge, MA, USA) used dilution 1:5000 overnight at 4. anti-ITPKB specific antibody recognizing 67.4 kDa band of ITPKB. The blots were washed three times for 20 min with TBS-T buffer and then incubated with horseradish peroxidase-conjugated secondary antibodies (1:5000–1:10000 dilution) for 1 h at room temperature. After washing three times for 20 min in TBS-T buffer, blots were developed using enhanced chemiluminescence reagents (ECL, Thomas Scientific, Rockford, IL, USA) and exposed to clear blue X-ray film.

#### DNA sequencing of ITPKB gene

Eight Exons and the adjacent introns of the *ITPKB* gene were amplified by PCR. *ITPKB* gene cloning and DNA sequencing primers are shown in Table 3. Exons 1, 2d, 2e, 3, 4, 5, 6, 7 and 8 of the *ITPKB* gene were amplified by PCR in a 25-μl reaction mixture containing 2 μl of 10 μM forward and reverse primers, 2 μl of 25 mM MgCl_2_, 2.5 μl of 10 × buffer, 1 μl of 10 mM dNTPs, 50 ng of genomic DNA and 0.2 μl of 1 μ μl^−1^ AmpliTaq Gold DNA polymerase (Life Technologies, Applied Biosystems). PCR conditions were as follows: 94 °C for 5 min, followed by 35 cycles of 95 °C for 30 s (s), 60 °C for 45 s, and 72 °C for 45 s with a final 10-min extension period at 72 °C. Certain regions of exons 2a, 2b and 2c are GC rich (74% of GC). These were amplified by a GC-rich PCR Kit (Roche Life Science) according to the manufacturer's protocol. Each 25 μl PCR reaction mixture contained 2 μl of 10 μm forward and reverse primers, 1 μl of 10 mM dNTPs, 2.5 μl of 5 m GC-Rich resolution solution, 5 μl of 5x GC-rich PCR reaction buffer and 0.5 μl of GC-rich PCR enzyme. PCR conditions were as follows: 94 °C for 3 min, followed by 10 cycles of 95 °C for 30 s, 60 °C for 45 s, and 72 °C for 45 s; followed by 20 cycles of 95 °C for 30 s, 60 °C for 45 s, and 72 °C for 50 s, with a final 7-min extension period at 72 °C.

PCR products were separated by electrophoresis and purified (Zymoclen gel DNA Recovery Kit, Zymo Research, Orange, CA, USA). The eight exons and adjacent intronic regions were sequenced by Retrogen, Inc. (San Diego, CA, USA). DNA sequences were analyzed for bi-directional sequence with DNASTAR Lasergene, Edit Sequence (DNASTAR, Inc., Madison, WI, USA) and the Sequence Scanner Software 1.0 (Life Technologies, Applied Biosystems).

## Figures and Tables

**Table 1:** Immunological features of a patient with CVID

	*Patient*	*Reference ranges*
*Lymphocyte subsets (%)*		
CD3+	744 (62%)	844–2395 (65–80%)
CD3+CD4+	504 (42%)	437–1597 (31–56%)
CD3+CD8+	204 (17%)	281–1125 (17–34%)
CD4+/CD8+ ratio	2.57	0.78–2.21
CD19+	60 (5%)	132–399 (4–16%)
CD3-CD56+CD16+	36 (3%)	120–424 (4–16%)

*Lymphocyte proliferation to antigens and mitogens (c.p.m.)*		
Mumps	42	2052–26 495
Tetanus toxoid	762	6092–94 539
*Candida albicans*	279	13 249–60 917
PPD	174	548–2580
PHA	83 013	114 881–289 206
Con A	97 975	131 199–252 925
PWM	15 320	20 171–78 728

*Serum immunoglobulins (mg dl^−1^)*		
IgG	516	694–1618
IgA	44	68–378
IgM	95	65–263
IgE (IU l^−1^)	110	10–150

*Specific antibodies*		
*Streptococcus pneumoniae* (μg ml^−1^, 14 serotypes)	0.0–0.3^a^	>1.3^b^

*Complement*		
CH50 (U ml^−1^)	267	(101–300)
C3 (mg dl^−1^)	113	(88–201)
C4 (mg dl^−1^)	31	(16–47)

*Oxidative respiratory burst (MFI)*		
Unactivated	54	70
PMA-activated	404	873
Ratio of activated/unactivated	7.5	12.5

Abbreviations: ConA, concanavalin A; CVID, common variable immunodeficiency; Ig, immunoglobulin; MFI, mean fluorescence intensity; PHA, phytohemagglutinin; PMA, phorbol myristate acetate; PPD, purified protein derivative; PWM, pokeweed mitogen.

a After Pneumovax-23 response. Patient did not respond to any of the 14 serotypes.

b 1.3 protective titers. × 4 fold increase following Pneumovax-23 against at least 70% serotypes.

**Table 2:** Allele frequencies of *ITPKB* reported by UCSC

dbSNP: rs6667260:
A: 44.423% (3664.890/8250); **C**: 55.577% (4585.110/8250)
Coding annotations by dbSNP:
ITPKB (NM_002221): missense variant S (**T**CC) → A (**G**CC)
dbSNP: rs708776:
**A**: 95.881% (9334.020/9735); C: 3.461% (336.980/9735); G: 0.637% (62/9735); T: 0.021% (2/9756)
Coding annotations by dbSNP:
ITPKB (NM_002221): missense variant P (C**C**G) → Q (C**A**G)
dbSNP: rs708775:
C: 71.623% (7217.720/10077.330); **T**: 28.377% (2859.610/10077.330)
Coding annotations by dbSNP:
ITPKB (NM_002221): synonymous variant A (GC**C**) → A (GC**T**)

Bold letter indicates the nucleotide of variance from patient.

**Table 3:** Primers for *ITPKB* gene cloning and sequencing

*Location*	*Forward (5*′→*3*′*)*	*Reverse (5*′→*3*′*)*	*Length*
Exon I	GGGCCCCTGAACCGAAGAGC	CGCCAGCAGAGCCGCCCCGAGAC	474
Exon II a	TTCTGCAGGCGACCCCCAACT	CTCAAACATGCCCACTTTCTGG	725
Exon II b	GCTATGCGCTCAATAGCCTG	CGGAGTCGGTTCCTGAAGTC	638
Exon II c	CCGGAGGAGGCCAAGAGGAAG	ACGGTGTGGCCCAGTGGATGTAAC	582
Exon II d1	AGTTACATCCACTGGGCCAC	AGATCTTTCGCAGCGTCCC	537
Exon II d2	GGGGGCGTTGGCAGCTCTCCGACAG	CCGGACTTGGGAGGGCATCACTGT	336
Exon II e	GGCAGAATGCTGGAGCCTT	CATGAGTTCTGGGCAAATCTCA	550
Exon III	CATAGGTGATGGTGGTGGCA	TCCAGAGAGGTCCGTTGTCTAT	450
Exon IV	GTGCTCAGTCCATAGCAGGG	GGAGGCTCTAATTGGTCCAACA	450
Exon V	TCGGAAAGACGTGCCTTG	CAAATGTCTGGCCTTGGG	500
Exon VI	AGGTTCAGATGGGGCTCG	GGATTACAGGCGTGAGCCAC	430
Exon VII	CTCTAGATGTCCGGCGTTTAGG	GTGCAGCGAGGGTCTGGT	343
Exon VIII	TGCAGGCACCCAACTGTC	GTAGGGTCCCCTCAGCAGC	670

## References

[bib1] 1Al-Herz W, Bousfiha A, Casanova JL, Chatila T, Conley ME, Cunningham-Rundles C et al. Primary immunodeficiency diseases: an update on the classification from the international union of immunological societies expert committee for primary immunodeficiency. Front Immunol 2014; 5: 162. 24795713 10.3389/fimmu.2014.00162 PMC4001072

[bib2] 2Ameratunga R, Brewerton M, Slade C, Jordan A, Gillis D, Steele R et al. Comparision of diagnostic criteria of common variable immunodeficiency disorder. Front Immunol 2014; 5: 415. 25309532 10.3389/fimmu.2014.00415 PMC4164032

[bib3] 3Park JH, Resnick ES, Cunningham-Rundles C. Perspectives on common variable Immune deficiency. Ann NY Acad Sci 2011; 246: 41–49. 10.1111/j.1749-6632.2011.06338.x PMC3428018 22236429

[bib4] 4Oksenhendler E, Gerard L, Fieschi C, Malphettes M, Mouillot G, Jaussaud R et al. Infections in 252 patients with common variable immunodeficiency. Clin Infect Dis 2008; 46: 1547–1554. 18419489 10.1086/587669

[bib5] 5Chapel H, Lucas M, Lee M, Bjorkander J, Webster D, Grimbacher B et al. Common variable immunodeficiency disorders: division into distinct clinical phenotypes. Blood 2008; 112: 277–286. 18319398 10.1182/blood-2007-11-124545

[bib6] 6Salzer U, Warnatz K, Peter HH. Common variable immunodeficiency-an update. Arthritis Res Ther 2012; 14: 223. 23043756 10.1186/ar4032 PMC3580506

[bib7] 7Baccelli C, Buckridge S, Thrasher A, Gasper HB. Molecular defects in common variable immunodeficiency. Clin Exp Immunol 2007; 149: 401–409. 17697196 10.1111/j.1365-2249.2007.03461.x PMC2219326

[bib8] 8Grimbacher B, Hutloff A, Schlesier M, Glocker E, Warnatz K, Dräger R et al. Homozygous loss of ICOS is associated with adult onset common variable immunodeficiency. Nat Immunol 2003; 4: 261–268. 12577056 10.1038/ni902

[bib9] 9Salzer U, Chapel HM, Webster AD, Pan-Hammarström Q, Schmitt-Graeff A, Schlesier M et al. Mutation in TNFRSF 13B encoding TACI are associated with common variable immunodeficiency in humans. Nat Genet 2005; 37: 820–828. 16007087 10.1038/ng1600

[bib10] 10Castigli E, Wilson SA, Garibyan L, Rachid R, Bonilla F, Schneider L et al. TACI is mutant in common variable immunodeficiency and IgA deficiency. Nat Genet 2005; 37: 829–834. 16007086 10.1038/ng1601

[bib11] 11Warnatz K, Salzer U, Rizzi M, Fischer B, Gutenberger S, Böhm J et al. B-cell activating factor receptor deficiency is associated with adult-onset antibody deficiency syndrome in human. Proc Natl Acad Sci USA 2009; 106: 13945–13950. 19666484 10.1073/pnas.0903543106 PMC2722504

[bib12] 12Losi CG, Silini A, Fiorini C, Soresina A, Meini A, Ferrari S et al. Mutational analysis of human BAFF-receptor (TNFRSF 13C (BAFF-R) in patients with common variable immunodeficiency. J Clin Immunol 2005; 25: 496–502. 16160919 10.1007/s10875-005-5637-2

[bib13] 13van Zelm MC, Reisli I, van der Burg M, Castaño D, van Noesel CJ, van Tol MJ et al. An antibody deficiency syndrome due to mutations in the CD19 gene. N Eng J Med 2006; 354: 1901–1912. 10.1056/NEJMoa051568 16672701

[bib14] 14Kuijpers TW, Bende RJ, Baars PA, Grummels A, Derks IA, Dolman KM et al. CD20 deficiency in humans results in impaired T cell independent antibody responses. J Clin Invest 2010; 120: 214–222. 20038800 10.1172/JCI40231 PMC2798692

[bib15] 15Thiel J, kimming L, Salzer U, Grudzien M, Lebrecht D, Hagena T et al. Genetic CD21 deficiency is associated with hypogammaglobulinemia. J Allergy Clin Immunol 2012; 129: 801–810. 22035880 10.1016/j.jaci.2011.09.027

[bib16] 16van Zelm MC, Smet J, Adams B, Mascart F, Schandené L, Janssen F et al. CD81 gene defect in human in humans disrupts CD19 complex formation and lead to antibody deficiency. J Clin Invest 2010; 120: 1265–1274. 20237408 10.1172/JCI39748 PMC2846042

[bib17] 17Lopez-Herrara G, Tampella G, Pan-Hammarstrom Q, Herholz P, Trujillo-Vargas CM, Phadwal K et al. Deleterious mutations in LRBA are associated with a syndrome of immune deficiency and autoimmunity. Am J Hum Genet 2012; 90: 986–1001. 22608502 10.1016/j.ajhg.2012.04.015 PMC3370280

[bib18] 18Choi KY, Kim HK, Lee SY, Moon KH, Sim SS, Kim JW et al. Molecular cloning and expression of a complimentary DNA for inositol 1,4,5 trisphosphate 3 kinase. Science 1990; 248: 64–66. 2157285 10.1126/science.2157285

[bib19] 19Dewaste V, Pouillon V, Moreau C, Shears S, Takazawa K, Erneux C. Cloning and expression of cDNA encoding human inositol 1,4,5, trisphosphate 3 kinase C. Biochem J 2000; 352: 343–351. 11085927 PMC1221465

[bib20] 20Hsu A-L, Ching T-T, Sen G, Wang DS, Bondada S, Authi KS et al. Novel function of phosphoinositide 3-kinase in T cell Ca2+ signaling. J Biol Chem 2000; 275: 16242–16250. 10748064 10.1074/jbc.M002077200

[bib21] 21Dewaste V, Moreau C, de Smedt F, Bex F, De Smedt H, Wuytack F et al. The isozymes of human inositol-1,4,5-trisphosphate 3-kinase show specific intracellular localization but comparable Ca2+ responses on transfection of COS-7 cells. Biochem J 2003; 374: 41–49. 12747803 10.1042/BJ20021963 PMC1223573

[bib22] 22Marechal Y, Pesesse X, Jia V, Pouillon V, Pérez-Morga D, Daniel J et al. Inositol 1,3,4,5-tetrakisphosphate controls proapoptotic Bim gene expression and survival in B cells. Proc Natl Acad Sci USA 2007; 104: 13978–13983. 17709751 10.1073/pnas.0704312104 PMC1955816

[bib23] 23Miller AT, Sanberg M, Huang YH, Young M, Sutton S, Sauer K et al. Production of Ins (1,3,4,5)P4 mediated by the kinase Itpkb inhibits store-operated calcium channels and regulate B cell selection and activation. Nat Immunol 2007; 8: 514–521. 17417640 10.1038/ni1458

[bib24] 24Schurmans S, Pouillon V, Marechal Y. Regulation of B cell survival, development and function by inositol 1,4,5,-trisphosphate-3 kinase B (itpkb). Adv Enzyme Regul 2011; 51: 66–73. 21035494 10.1016/j.advenzreg.2010.08.001

[bib25] 25Miller AT, Beisner DR, Liu D, Cooke MP. Inositol 1,4,5-trisphosphate 3-kinase B is a negative regulator of BCR signaling that controls B cell selection and tolerance induction. J Immunol 2009; 182: 4696–4704. 19342645 10.4049/jimmunol.0802850

[bib26] 26Jia Y, Loison F, Hattori H, Li Y, Erneux C, Park SY et al. Inositol trisphosphate 3-kinase B (InsP3KB) as a physiological modulator of myelopoiesis. Proc Natl Acad Sci USA 2008; 105: 4739–4744. 18339802 10.1073/pnas.0800218105 PMC2290809

[bib27] 27Jia Y, Subramanian KK, Erneux C, Pouillon V, Hattori H, Jo H et al. Inositol 1,3,4,5-tetrakisphosphate negatively regulates phosphatidylinositol-3,4,5-trisphosphate signaling in neutrophils. Immunity 2007; 27: 453–467. 17825589 10.1016/j.immuni.2007.07.016 PMC2084373

[bib28] 28Tajouri L, Mellick AS, Tourtellotte A, Nagra RM, Griffiths LR. An examination of MS candidate genes identified as differentially regulated in multiple sclerosis plaque tissue, using absolute and comparative real-time Q-PCR analysis. Brain Res Brain Res Protoc 2005; 15: 79–91. 15905117 10.1016/j.brainresprot.2005.04.003

[bib29] 29Emilsson L, Saetre P, Jazin E. Alzheimer's disease: mRNA expression profiles of multiple patients show alterations of genes involved with calcium signaling. Neurobiol Dis 2006; 21: 618–625. 16257224 10.1016/j.nbd.2005.09.004

[bib30] 30Györffy B, Lage H. A Web-based data warehouse on gene expression in human malignant melanoma. J Invest Dermatol 2007; 127: 394–399. 16946712 10.1038/sj.jid.5700543

[bib31] 31Finck A, Van den Meer JW, Scheffer AA, Pfannstiel J, Fieschi C, Plebani A et al. Linkage of autosomal dominant common variable immunodeficiency to chromosome 4q. Eur J Hum Genet 2006; 14: 867–875. 16639407 10.1038/sj.ejhg.5201634

[bib32] 32Fuchs HB, Slater L, Novey H, Ong K, Gupta S. Immunological analysis in familial common variable immunodeficiency. Clin Exp Immunol 1984; 56: 29–33. 6609034 PMC1535955

[bib33] 33Kralovicova J, Hammarstrom L, Plebani A, Webster AD, Voreschovsky I. Fine scale mapping at IGAD1 and genome-wide genetic linkage analysis implicate HLA-DQ/DR as a major susceptibility locus in selective IgA deficiency and common variable immunodeficiency. J Immunol 2003; 170: 2765–2775. 12594308 10.4049/jimmunol.170.5.2765

[bib34] 34Schroeder HW Jr, Zhu ZB, March RE, Campbell RD, Berney SM, Nedospasov SA et al. Susceptibility locus for IgA deficiency and common variable immunodeficiency in HLA-DR3, -B8, -A1 haplotype. Mol Med 1998; 4: 72–86. 9508785 PMC2230309

[bib35] 35Orange JS, Glessner JT, Resnick E, Sullivan KE, Lucas M, Ferry B et al. Genome-wide association identifies diverse causes of common variable immunodeficiency. J Allergy Clin Immunol 2011; 127: 1360–1367. 21497890 10.1016/j.jaci.2011.02.039 PMC3646656

[bib36] 36Giovanneti A, Pierdominici M, Mazzatta F, Marziali M, Renzi C, Mileo AM et al. Unrevelling the complexity of T cell abnormalities in common variable immunodeficiency. J Immunol 2007; 178: 3932–3943. 17339494 10.4049/jimmunol.178.6.3932

[bib37] 37Melo KM, Carvelho KI, Bruno FR, Ndhlovu LC, Ballan WM, Nixon DF et al. A decreased frequency of regulatory T cells in patients with common variable immunodeficiency. PLos ONE 2009; 4: e6269. 19649263 10.1371/journal.pone.0006269 PMC2715881

[bib38] 38Horn J, Manguiat A, Berglund LJ, Knerr V, Tahami F, Grimbacher B et al. Decrease in phenotypic regulatory T cells in subsets of patients with common variable immunodeficiency. Clin Exp Immunol 2009; 156: 446–454. 19438597 10.1111/j.1365-2249.2009.03913.x PMC2691973

[bib39] 39Yu GP, Chiang D, Song SJ, Hoyte EG, Huang J, Vanishsarn C et al. Regulatory T cell dysfunction in subjects with common variable immunodeficiency complicated by autoimmune disease. Clin Immunol 2009; 131: 240–253. 19162554 10.1016/j.clim.2008.12.006 PMC5140037

[bib40] 40Arumugakani G, Wood PM, Carter CR. Frequency of Treg is resuced in CVID patients with autoimmunity and splenomegaly and is associated with expanded CD21^lo^ B lymphocytes. J Clin Immunol 2010; 30: 292–300. 19997968 10.1007/s10875-009-9351-3

[bib41] 41Nalaskowski MM, Fliegert R, Ernst O, Brehm MA, Fanick W, Windhorst S et al. Human inositol 1,4,5-trisphosphate 3-kinase isoform B (IP3KB) is a nucleocytoplasmic shuttling protein specifically enriched at cortical actin filaments and at invaginations of nuclear envelop. J Biol Chem 2011; 286: 4500–4510. 21148483 10.1074/jbc.M110.173062 PMC3039344

[bib42] 42Warnatz K, Denz A, Drager R, Braun M, Groth C, Wolff-Vorbeck G et al. Severe deficiency of switched memory B cells (CD27+IgM-IgD-) in subgroups of patients with common variable immunodeficiency: a new approach to classify a heterogeneous disease. Blood 2002; 99: 1544–1551. 11861266 10.1182/blood.v99.5.1544

[bib43] 43Piqueras B, Lavenu-Bombled C, Galicier L, Bergeron-van der Cruyssen F, Mouthon L, Chevret S et al. Common variable immunodeficiency patient classification based upon impaired B cell memory differentiation correlates with clinical aspects. J Clin Immunol 2003; 23: 385–400. 14601647 10.1023/a:1025373601374

[bib44] 44Kimchi-Sarfaty C, Oh JM, Kim I-W, Sauna ZE, Calcagno AM, Ambudkar SV et al. A "silent" polymorphism in the MDR1 gene changes substrate specificity. Science 2007; 315: 525–528. 17185560 10.1126/science.1135308

[bib45] 45Chamary JV, Parmley JL, Hurst LD. Hearing silence: non-neutral evolution at synonymous sites in mammals. Nat Rev Genet 2006; 7: 98–108. 16418745 10.1038/nrg1770

[bib46] 46Goymer P. Synonymous mutations break their silence. Nat Rev Genet 2007; 8: 92.

[bib47] 47Zhou T, Ko EA, Gu W, Lim I, Bang H, Ko JH. Non-silent story on synonymous sites in voltage-gated ion channel genes. PLoS ONE 2012; 7: e48541. 23119053 10.1371/journal.pone.0048541 PMC3485311

[bib48] 48Carlini DB, Stephan W. *In vivo* introduction of unpreferred synonymous codons into the Drosophila Adh gene results in reduced levels of ADH protein. Genetics 2003; 163: 239–243. 12586711 10.1093/genetics/163.1.239 PMC1462401

[bib49] 49Carlini DB. Experimental reduction of codon bias in the Drosophila alcohol dehydrogenase gene results in decreased ethanol tolerance of adult flies. J Evol Biol 2014; 17: 779–785. 10.1111/j.1420-9101.2004.00725.x 15271077

[bib50] 50Pagani F, Raponi M, Baralle FE. Synonymous mutations in CFTR exon 12 affect splicing and are not neutral in evolution. Proc Natl Acad Sci USA 2005; 102: 6368–6372. 15840711 10.1073/pnas.0502288102 PMC1088389

[bib51] 51Chubb JE, Bradshaw NJ, Soares DC, Porteous DJ, Millar JK. The DISC locus in psychiatric illness. Mol Psychiatry 2008; 13: 36–64. 17912248 10.1038/sj.mp.4002106

[bib52] 52Zhao Y, Loyer P, Li H, Valentine V, Kidd V, Kraft AS. Cloning and chromosomal location of a novel member of the myotonic dystrophy family of protein kinases. J Biol Chem 1997; 272: 10013–10020. 9092543 10.1074/jbc.272.15.10013

[bib53] 53Cmejla R, Petrak J, Cmejlova J. A novel iron responsive element in the 3'UTR of human MRCKalpha. Biochem Biophys Res Commun 2006; 341: 158–166. 16412980 10.1016/j.bbrc.2005.12.155

